# Genome-wide characterization of hypothiocyanite stress response in *Escherichia coli*

**DOI:** 10.1128/jb.00524-24

**Published:** 2025-04-29

**Authors:** Julia D. Meredith, Michael J. Gray

**Affiliations:** 1 Department of Microbiology, School of Medicine,The University of Alabama at Birmingham 318277, Birmingham, Alabama, USA; University of Virginia School of Medicine, Charlottesville, Virginia, USA

**Keywords:** oxidative stress

## Abstract

Oxidative stress is one of the major methods of microbial population control and pathogen clearing by the mammalian immune system. The methods by which bacteria are able to escape damage by host-derived oxidants such as hydrogen peroxide (H_2_O_2_) and hypochlorous acid (HOCl) have been relatively well described, while other oxidants’ effects on bacteria and their genetic responses are not as well understood. Hypothiocyanite/hypothiocyanous acid (OSCN^–^/HOSCN) is one such oxidative stress agent. In this study, we used RNA-sequencing to characterize the global transcriptional response of *Escherichia coli* to treatment with HOSCN and the impact of deletions of the HOSCN resistance proteins RclA (HOSCN reductase), RclB, and RclC on that response. The HOSCN response of *E. coli* was different from the previously characterized responses of *E. coli* to other oxidants such as H_2_O_2_, superoxide, or HOCl and distinct from the reported responses of other bacteria such as *Streptococcus pneumoniae* and *Pseudomonas aeruginosa* to HOSCN. Strikingly, deletion of any one of the Rcl proteins had very similar effects on the transcriptional response to HOSCN, indicating that any disruption of HOSCN defense in *E. coli* results in similar impacts, despite the fact that we do not currently understand the mechanism(s) by which RclB and RclC contribute to that defense.

**IMPORTANCE:**

Understanding how bacteria sense and respond to oxidative stress provides insights into how our bodies interact with the microbial population within us. In this study, we have characterized the genetic response of *E. coli* to the important immune oxidant hypothiocyanite and investigated the role of *rclABC* genes in that response.

## INTRODUCTION

The innate immune system uses a variety of systems to control the population of microbes on epithelial surfaces (1). Understanding how some bacteria, whether commensal or pathogenic, are able to evade inhibition by the immune system while others are not is broadly important for human health (2). Among the tools available to the innate immune system is the production of reactive oxidants that inhibit bacterial growth (3, 4). Hypothiocyanite/hypothiocyanous acid (OSCN^–^/HOSCN) is one such oxidant (5–7). It is related to hypohalous acids such as hypochlorous and hypobromous acid (HOCl and HOBr, respectively) (8). HOSCN can be formed by three different heme peroxidase enzymes in the human body: lactoperoxidase (LPO), found secreted into multiple fluids, including saliva and breastmilk; eosinophil peroxidase (EPO), produced by eosinophils; and myeloperoxidase (MPO), which is found in leukocytes (9–12). These peroxidase enzymes catalyze the reaction between H_2_O_2_ and thiocyanate (SCN^–^) to form HOSCN (10).

HOSCN almost exclusively oxidizes thiols (13). In bacterial cells, this means that the impact of HOSCN occurs on cysteine-containing proteins, glutathione, and other low-molecular weight thiols (14). Treatment with HOSCN causes the formation of sulfenic acids and disulfide bonds, which leads to the inhibition of many central cellular processes and potentially to protein aggregation (8, 12, 14).

The relationship between the immune system-produced antimicrobials and the microbiome is complex. While bacterial response to oxidative stress is not a new field of study, there is still much unknown about how the bacteria differentially respond to the various oxidants in the human body. The transcriptional response of *E. coli* to H_2_O_2_ is different from its response to HOCl (15, 16), and the response of *Streptococcus pneumoniae* to H_2_O_2_ is different from that of *E. coli* (15, 17). While HOSCN as an antimicrobial product of LPO has been studied since as early as the 1970s (18, 19), advances in how bacteria sense and respond to it have only come about in recent years (20–22). In fact, for a long time, HOSCN was considered a highly specific inhibitor of bacteria because mammalian cells are able to use a selenocysteine-containing thioredoxin reductase enzyme (sec-TrxR) to reduce HOSCN to SCN^–^ and water (23), while bacterial cells were thought to not possess a specific defense mechanism against it. Recently, however, we discovered that the *rcl* operon of *E. coli* encodes an efficient bacterial HOSCN reductase, which we call RclA (20), indicating that this assumption was not true and that bacteria can mount a defense against HOSCN stress.

In *E. coli*, the *rcl* operon is controlled by the transcriptional regulator RclR, which upregulates three genes in response to HOSCN: *rclA* and two additional genes called *rclB* and *rclC*, which are conserved in the *Enterobacteriaceae* family, but whose function is unknown. RclB is a small periplasmic protein, and RclC is an inner-membrane protein (6, 20). In this study, we examined the overall transcriptional response of *E. coli* to treatment with HOSCN, revealing that it is markedly different from the characterized responses of other bacteria to HOSCN, or of *E. coli* to other oxidants. We also observed that when any of the *rcl* genes are deleted, the transcriptional response of *E. coli* to HOSCN becomes more drastic, which provided us new insights into how *rclB* and *rclC* may help protect from HOSCN stress. The permeability of membranes to specific oxidants has a large effect on how those oxidants affect bacteria (24). Based on the transcriptional impacts and protein homology, we hypothesized that RclB might function by impacting the permeability of the *E. coli* outer membrane to HOSCN, but this does not appear to be the case, leaving the mechanism(s) by which RclB and RclC contribute to the coordinated HOSCN defense system still unknown.

## RESULTS AND DISCUSSION

### The transcriptional response of *E. coli* to HOSCN differs from other stress responses and from the known responses of other bacteria to HOSCN

Bacterial responses to important cellular oxidants such as H_2_O_2_ and other reactive oxygen species (ROS) have been well characterized (15, 25), as have the responses to reactive chlorine species such as HOCl (16). HOSCN reductases (RclA and its homologs Har and MerA) have recently been shown to be important in HOSCN stress resistance in *E. coli*, *Streptococcus pneumoniae*, and *Staphylococcus aureus* (20–22, 26, 27), and mutants of *E. coli* and *S. pneumoniae* lacking glutathione oxidoreductase (Gor) are very sensitive to HOSCN (21, 26, 28), but limited research has been done to explore the broader bacterial response to HOSCN or the impact of the RclA, RclB, and/or RclC proteins on that response. The entire transcriptional response to stress caused by HOSCN has only been studied in *Pseudomonas aeruginosa* strain PAO1 (29, 30), which lacks homologs of any of the *E. coli* Rcl proteins (31), and transposon sequencing has been used to identify mutants with altered HOSCN sensitivity in *S. pneumoniae* (32), which encodes only the RclA homolog Har and no homologs of RclB or RclC (21, 31).

**Fig 1:**
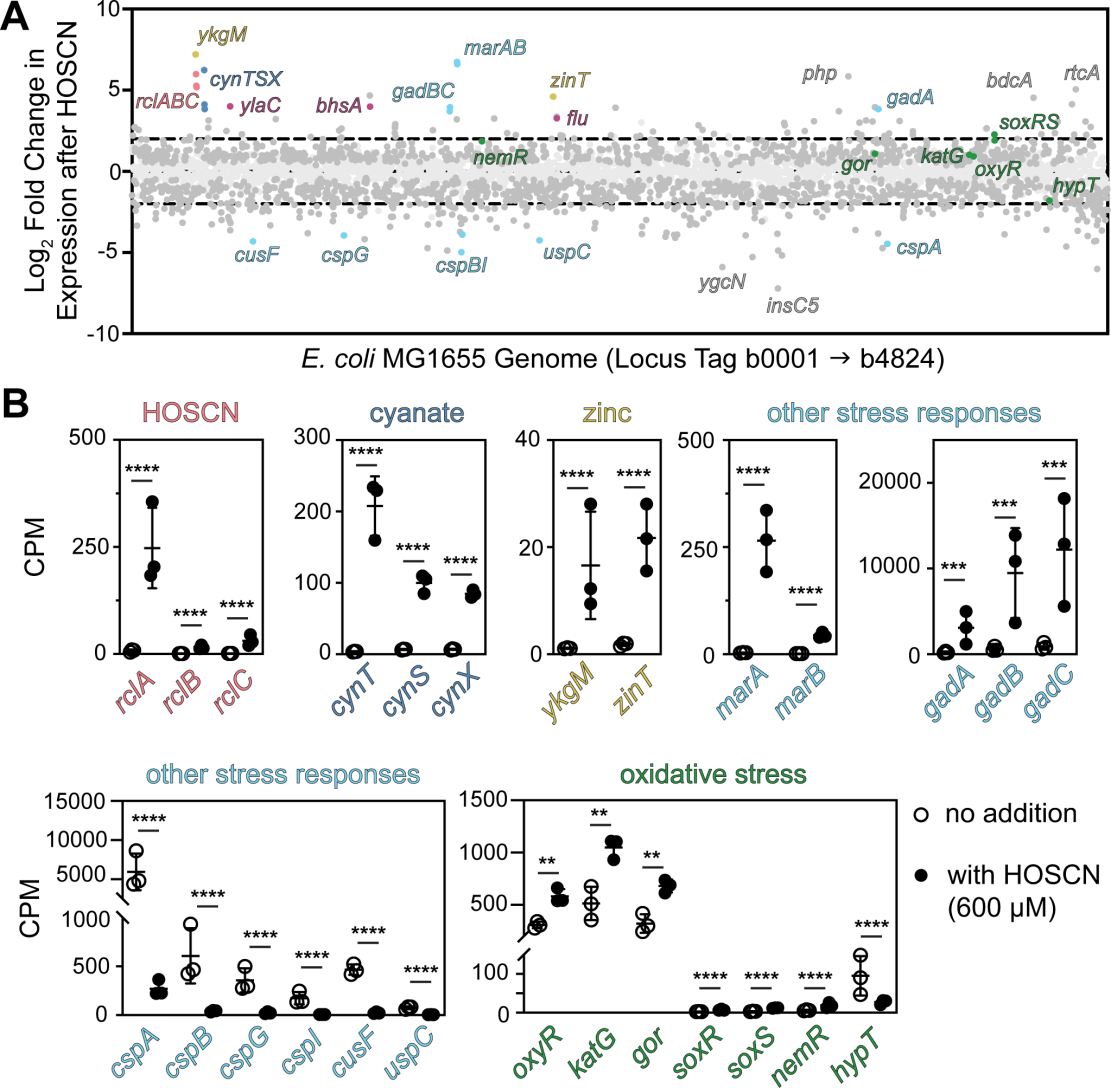
The transcriptional response to HOSCN in *E. coli*. (**A**) Transcriptomic response of *E. coli* MG1655 treated with 600 µM HOSCN compared to untreated *E. coli*. Each dot represents a gene, organized in order on the X-axis. Any gene above or below the dashed line had a log-fold change greater than 2 or −2, respectively. (**B**) The indicated genes of interest were taken from the same data set and graphed by normalized counts per million with and without HOSCN treatment. Statistical significance calculated in Prism GraphPad by two-way ANOVA of log-transformed CPM + 1 values, with significant differences in gene expression with and without HOSCN indicated: ** =*P*-value <0.01, ***= *P*-value <0.001, and ****= *P*-value <0.0001. Full RNA-seq results (**A**) are in Dataset S1, and complete ANOVA results (**B**) are in Table S1. A heatmap of the transcriptomics results can be found as Fig. S1.

**Fig 2:**
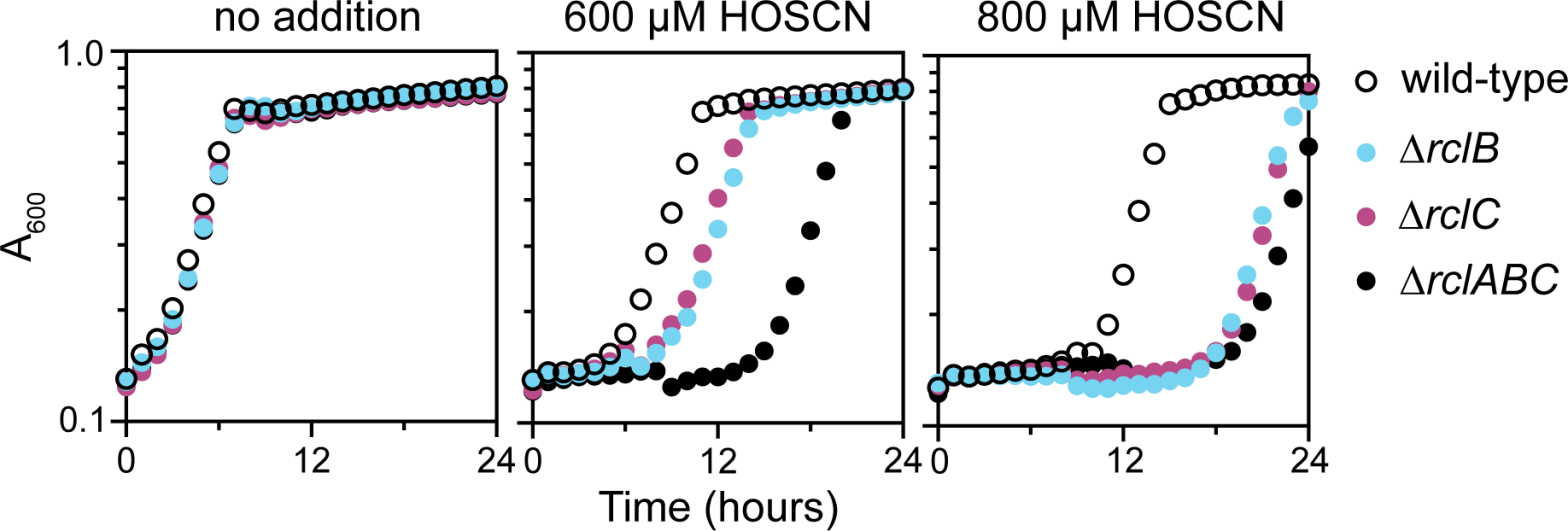
RclB and RclC are required for HOSCN stress resistance in *E. coli*. *E. coli* MG1655 wild-type, MJG0047 (MG1655 ∆*rclB*), MJG0013 (MG1655 ∆*rclC::cat*^+^), and MJG0901 (MG1655 ∆*rclABC*) were inoculated into M9 minimal medium containing the indicated concentrations of HOSCN and incubated at 37°C with shaking for 24 hours in a Tecan Sunrise plate reader, measuring A_600_ every 30 minutes. Each graph shows the mean of four technical replicates, and two additional experimental replicates are shown in Fig. S1.

We therefore measured the transcriptomic activity of *E. coli* strain MG1655 with and without treatment with 600 µM HOSCN by RNA sequencing (Fig. 1A; Dataset S1; Fig. S1). This dose was chosen because it has a moderate inhibitory effect on the growth of wild-type *E. coli* in minimal medium (Fig. 2), while still permitting full recovery. No killing of *E. coli* was observed at this dose (Fig. S2). While this is likely a higher bolus addition of HOSCN than bacteria encounter in host environments, where the highest measured concentrations are a steady state of ~70 µM in saliva (33–35), to our knowledge, no measurements of HOSCN concentrations in the intestine (where *E. coli* naturally resides), either globally or in inflamed microenvironments, have been reported. It is also comparable to the doses of HOSCN necessary for inhibition of other bacteria in pure culture (6, 22, 29, 30, 36). Notably, *S. pneumoniae* requires a bolus addition of 800 µM HOSCN to see the effects of deletion of the RclA homolog of that species on growth inhibition in pure culture (21), but that same mutant is profoundly defective in host colonization (27). Our *E. coli* transcriptomic results showed the expected strong upregulation of the *rclABC* genes (*P* < 0.0001) (Fig. 1B) (20). However, while the log-fold change in *rclB* and *rclC* expressions was statistically significant (*P* < 0.0001) and fairly substantial (around 3.5 fold and 4.5 fold, respectively), the amount of RNA transcribed for each of these genes was about tenfold lower than that of *rclA*, despite the fact that these three genes are thought to be in an operon controlled by the RclR transcription factor acting at the *rclA* promoter (37, 38). This suggests that there is more complexity to the transcriptional regulation of the *rcl* operon than previously appreciated.

**Fig 3:**
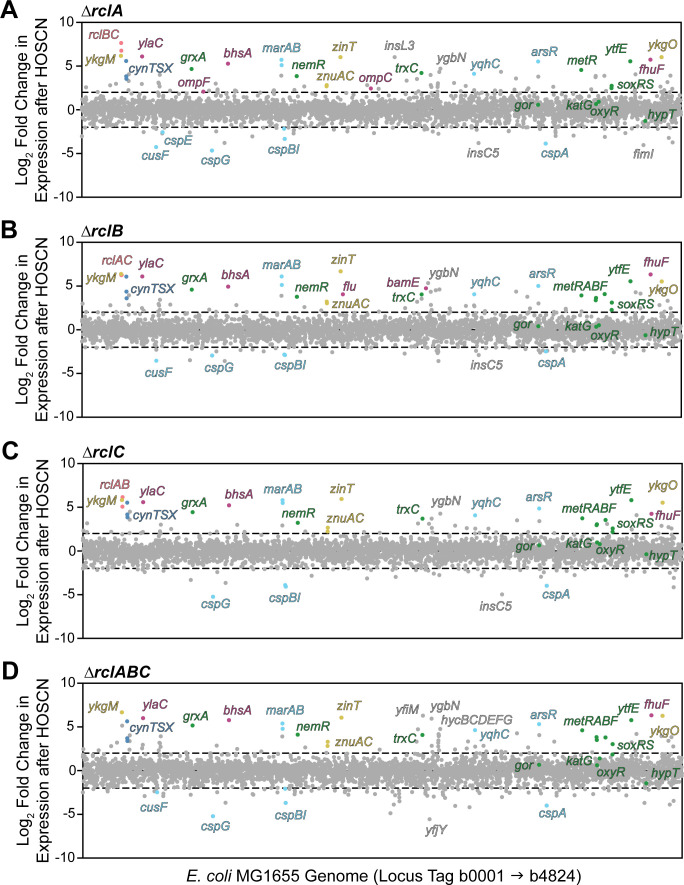
The transcriptional response of *E. coli* ∆*rclA*, ∆*rclB*, ∆*rclC*, and ∆*rclABC* mutants to HOSCN. *E. coli* transcriptional response to 600 µM HOSCN in strains (**A**) MJG1958 (MG1655 ∆*rclA*), (**B**) MJG0047 (MG1655 ∆*rclB*), (**C**) MJG0013 (MG1655 ∆*rclC::cat*^+^), or (**D**) MJG0901 (MG1655 ∆*rclABC*). Each dot represents a gene, organized in order on the X-axis. Any gene above or below the dashed line had a log-fold change greater than 2 or −2, respectively. Full RNA-seq results are in Dataset S1, and a heatmap can be found as Fig. S1.

**Fig 4:**
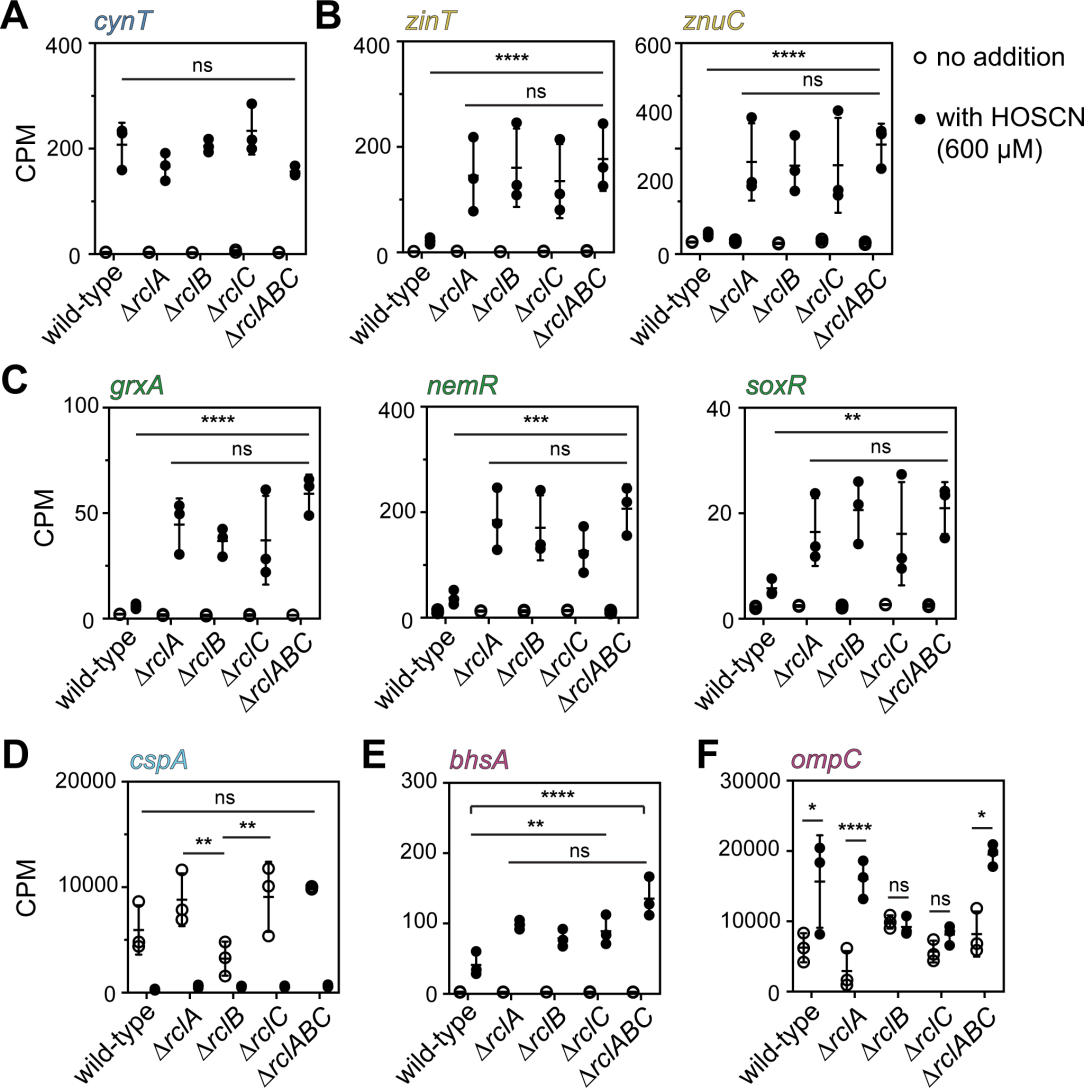
Expression of selected genes, illustrating notable features of the transcriptional response of *E. coli* ∆*rclA*, ∆*rclB*, ∆*rclC*, and ∆*rclABC* mutants to HOSCN. CPM values with and without 600 µM HOSCN treatment (see Fig. 1 and 3) for (**A**) the cyanate response gene *cynT*; (**B**) the zinc starvation response genes *zinT* and *znuC*; (**C**) the redox stress response genes *grxA, nemR*, and *soxR*; (**D**) the cold shock gene *cspA*; (**E**) the copper resistance gene *bhsA*; and (**F**) the *ompC* outer membrane porin gene in strains MG1655, MJG1958 (MG1655 ∆*rclA*), MJG0047 (MG1655 ∆*rclB*), MJG0013 (MG1655 ∆*rclC::cat*+), and MJG0901 (MG1655 ∆*rclABC*). Statistical significance calculated in Prism GraphPad by two-way ANOVA of log-transformed CPM + 1 values, with significant differences of interest indicated: ns = not significant, * = *P*-value <0.05; ** =*P*-value <0.01; *** = *P*-value <0.001; **** = *P*-value <0.0001. Note that panel (**F**) is the only one in this figure that shows comparisons between the expression with and without HOSCN, all of which were significant for every strain in all of the other panels. Full ANOVA results in Table S1.

Other results of this experiment revealed novel aspects of the HOSCN stress response in *E. coli*, which overlaps only tangentially with the well-characterized responses to other oxidants (15, 16, 25). For example, we observed 10- to 50-fold upregulation (*P* < 0.0001) of the *cynTSX* operon (Fig. 1B), which encodes genes involved in the transport and detoxification of cyanate (OCN^–^) (39). Expression of this operon is controlled by the OCN^–^-responsive transcription factor CynR (40), but whether this activation is due to HOSCN itself, to recognition by CynR of the SCN^-^ present in our HOSCN solution (6, 26), or to OCN^–^ contamination in our SCN^–^ solution is unclear. Treatment of *E. coli* with SCN^–^ did lead to induction of *cynT* expression (Fig. S3). This, in combination with results shown below (Fig. 3 and 4), led us to conclude that one of the second two possibilities was most likely. Genes involved in cyanate detoxification were upregulated in one study of HOSCN response in *P. aeruginosa* (29), but not the other (30).

A probably more physiologically relevant response we observed suggested that *E. coli* experiences zinc depletion upon treatment with HOSCN (Fig. 1B), with about tenfold upregulation (*P* < 0.0001) of *ykgM*, encoding a zinc-free alternative 50S ribosomal subunit protein L31B (41), and of *zinT*, encoding a periplasmic zinc-binding protein, both of which are known to be upregulated during zinc limitation (42, 43). Zinc status in *E. coli* is sensed by the transcription factor Zur, and oxidation of the zinc-binding cysteines in this protein by HOSCN could mimic the effect of low cytoplasmic zinc concentrations (44, 45). It is also possible that the release of zinc from HOSCN-oxidized zinc-binding proteins (e.g. Zur and Hsp33) (46, 47) could actually increase cytoplasmic zinc levels. We have previously reported that RclA is sensitive to inhibition by zinc *in vitro* (26), and it is certainly possible that dysregulation of zinc levels has impacts on *E. coli* survival under HOSCN stress conditions. However, *P. aeruginosa* does not show any transcriptional signatures of zinc limitation under HOSCN stress (29, 30).

Genes involved in the general stress response (Fig. 1B) that were upregulated 10 to 100-fold under HOSCN treatment include the multiple antibiotic resistance genes *marA* and *marB* (*P* < 0.0001), which are implicated in responses to many different kinds of stress (48), as well as the acid-stress resistance genes *gadABC* (49)(*P* < 0.001). Some other general stress response genes were significantly downregulated (*P* < 0.0001), under treatment, notably including the *csp* family of genes involved in the cold-shock response (50, 51), as well as *cusF*, encoding a periplasmic copper- and silver-binding protein (52), and *uspC*, encoding a so-called universal stress protein implicated in resistance to UV radiation and salt stress (53). It is not immediately clear what role any of these responses might play in HOSCN stress responses, although the results shown below (Fig. 3 and 4) do help clarify some of those results. Again, none of these responses are mirrored in the transcriptomic results in HOSCN-stressed *P. aeruginosa* (29, 30).

Comparison of the HOSCN response to other oxidative stress responses of *E. coli* revealed only limited overlaps (Fig. 1B) (15, 16, 25). The genes encoding the H_2_O_2_-sensing transcription factor OxyR and its regulon, represented here by *katG*, encoding catalase, and *gor*, encoding glutathione oxidoreductase (25), were significantly (*P* < 0.01) upregulated by HOSCN, albeit by less than twofold, despite how important we know that Gor is to HOSCN survival in both *E. coli* and *S. pneumoniae* (26, 32). The superoxide stress response genes *soxR* and *soxS* (25) were both significantly upregulated by HOSCN (threefold to sixfold, *P* < 0.0001), but the levels of mRNA for each of these genes were very low under both conditions. The gene encoding the transcriptional repressor NemR, which is important to *E. coli*’s response to HOCl (54), was only slightly upregulated in response to HOSCN (twofold, *P* < 0.0001), while the gene encoding another transcription factor implicated in the HOCl response, *hypT* (55), was slightly downregulated (fourfold, *P* < 0.0001). In *P. aeruginosa*, Groitl et al. (30) observed an upregulation of *nemR* in response to HOSCN, but Farrant et al. (29) did not. In contrast, only Farrant et al. observed upregulation of catalase and superoxide dismutase (29). Neither study reported up- or downregulation of *hypT* (29, 30). Consistent with the results of both *P. aeruginosa* studies (29, 30), though, we also did not observe substantial upregulation of chaperones or other heat-shock proteins, indicating that HOSCN does not cause substantial protein aggregation in either species, unlike HOCl (56).

We also did observe about 15-fold HOSCN-dependent upregulation of *bhsA* (Fig. 1A; Dataset S1) (also known as *comC*), which encodes a small periplasmic / outer membrane protein that impacts biofilm formation and appears to modify the permeability of the outer membrane to copper by an unknown mechanism, playing a role in increasing resistance to copper toxicity (57, 58). We will return to this gene in the sections below (Fig. 3, 4 and 6) for a more thorough discussion of the implications of this result.

Finally, a variety of other genes with poorly characterized functions were notably up- or downregulated as well, but what role these genes might play in HOSCN response is not clear. These included the genes encoding the putative inner membrane protein YlaC (59), the putative zinc-dependent hydrolase Php (60), the c-di-GMP binding protein BdcA (61), the RNA 3'-terminal phosphate cyclase RtcA (62), and the putative oxidoreductase YgcN (59).

### Knockouts of *rcl* genes are more sensitive to HOSCN than wild-type *E. coli*

The *rclABC* operon of *E. coli* is of particular interest to our group (20, 26, 31, 38). The functions of RclB and RclC*,* encoded in an operon downstream of *rclA* and mostly conserved only among members of the Enterobacteriaceae (31, 38), have not been elucidated. RclB is a predicted periplasmic protein belonging to the DUF1471 family of protein domains of unknown function (63, 64), and RclC is a predicted inner membrane protein (38) of the DUF417 family (63), but little else is known about their functions. It is important to note that neither RclB nor RclC possesses cysteine residues and therefore are unlikely to be oxidized directly by HOSCN (13). An RclC homolog called RcrB, which is found in uropathogenic *E. coli,* plays an important and specific role in HOCl resistance, but its mechanism of action has also not been characterized (65). Null mutations in *rclB* or *rclC* confer substantial sensitivity to HOSCN, comparable to the very high sensitivity of an ∆*rclABC* triple mutant (26) at high HOSCN concentrations (Fig. 2; Fig. S4). Notably, the sensitivities of ∆*rclB* and ∆*rclC* mutants were very similar to one another under all tested conditions, suggesting that they may function together.

### *E. coli* mounts a specific response to HOSCN when *Rcl* genes are mutated

*E. coli*’s response to HOSCN was very noticeably different when any of the *rcl* genes are mutated when compared to the wild-type (Fig. 3; Fig. S1). However, surprisingly, the response between individual mutations was not extremely variable, suggesting that RclA, RclB, and RclC form a coordinated defense against HOSCN and that the loss of any one of these proteins leads to similar breakdowns in the ability of the cell to respond appropriately to HOSCN stress, at least at a transcriptional level. Across the board, we observed stronger upregulation of genes involved in the metal stress response, envelope stress response, methionine synthesis, and oxidative stress response when any of the *rcl* genes was disrupted. In Fig. 4, we present a set of representative genes of interest in more detail to compare the induction by HOSCN in the wild-type and each mutant strain.

Many of the general stress response genes that were upregulated in the general response of *E. coli* were equally upregulated in the *rcl* mutant strains, including *cynT* (Fig. 4A), indicating a consistent OCN^–^ stress response that was not dependent on the *rcl* operon. This reinforced our conclusion that the expression of *cynTSX* was not likely to be a *bona fide* HOSCN response. Similarly, for example, the *marA* and *marB* genes, encoding regulators of genes involved in responses to a variety of toxic compounds (48), notably those encoding the AcrAB efflux pump (66), were equally strongly upregulated by HOSCN, regardless of the presence of *rclABC* (Fig. 1 and 3; Dataset S1). However, *acrA* and *acrB* were not substantially upregulated in any HOSCN-treated strain (Dataset S1), making the significance of *marAB* upregulation unclear.

More commonly, we observed stress response genes that were much more strongly upregulated in all four *rcl* mutant strains than in the wild-type, but which were not significantly different among the mutant strains (Fig. 4B and C). For example, the zinc starvation response, which was seen in wild-type (Fig. 1B), is much more evident under mutated *rcl* conditions (Fig. 4B), as demonstrated by the nearly tenfold greater upregulation of *zinT* and the fivefold to tenfold upregulation of *znuC*, which encodes part of an ABC transporter that is involved in zinc uptake (43, 67). There is also a much more prominent signature of oxidative stress response to HOSCN in the *rcl* mutant strains. For example, expression of *grxA,* encoding one of the four glutaredoxins found in *E. coli* (68), is induced by three to fivefold in the *rcl* mutants relative to less than twofold in the wild-type (Fig. 4C), which was somewhat surprising, considering that we have recently reported that mutants of *E. coli* lacking *grxA* are not more sensitive to HOSCN than the wild-type (26). Because of HOSCN’s ability to efficiently oxidize low-molecular weight thiols (13, 69, 70), the connection between glutathione and bacterial resistance to HOSCN has recently been of interest in the field. *E. coli* ∆*gor* mutants (lacking glutathione oxidoreductase) (59, 71), for instance, are highly sensitive to HOSCN (26). In *S. pneumoniae*, double mutants of *gor* and *har* (encoding the RclA homolog of that species) (21) are also highly sensitive to HOSCN (21, 28), and indeed we did see a general upregulation of *gor* in *E. coli* under HOSCN treatment. However, this was consistently a twofold induction or less and was not substantially affected by mutation of the *rcl* genes (Fig. 1 and 4; Dataset S1).

When *E. coli* is exposed to HOCl stress, there is strong upregulation of the redox-sensitive transcription factor-encoding gene *nemR* (54). Under HOSCN stress, we observed only a modest (~ twofold) upregulation of *nemR* in the wild-type (Fig. 1B), with a much stronger (fivefold to tenfold) upregulation when any or all of the three *rcl* genes are missing (Fig. 4C). Likewise, *soxR,* encoding a transcription factor that responds to superoxide, redox-cycling drugs, and nitric oxide (48, 72), is expressed in two to threefold higher amounts in *rcl* mutants (Fig. 4C). NemR is a bleach-sensing transcriptional regulator that responds to HOCl stress through oxidation of its cysteine residues (54, 73), and SoxR senses various oxidative stresses via an oxidation-sensitive cysteine-based iron-sulfur cluster (72). Logically, it makes sense that there would be more protein cysteine oxidation in a strain of *E. coli* missing specific defenses against HOSCN (13, 69, 70). The upregulation of the MetJ regulon (74) in *rcl* mutants (Fig. 3; Dataset S1) suggests that methionine may also be oxidized under these conditions, although this was unexpected since HOSCN does not itself oxidize methionine (13). It is conceivable that the metabolic stress of HOSCN exposure leads to dysregulation of methionine sulfoxide repair pathways and causes methionine sulfoxide to accumulate, but this remains speculative. It is interesting, however, that across the board, there is little difference in the amount of upregulation of oxidative stress responses in individual mutants. Mutants lacking *rclB* or *rclC* are not as sensitive to HOSCN as a mutant of *rclA* (20) or as the triple knockout (Fig. 2), and *E. coli* does not transcribe as much *rclB* or *rclC* as *rclA* mRNA (Fig. 1B) (although we do not know at this time if this is reflected in relative protein abundances), but the different mutant cells seem to be perceiving the same amount of overall oxidative stress, based on their transcriptional responses.

The impact of *rcl* mutations on the downregulated *csp* genes (Fig. 1B) was less consistent. For example, while there was no significant difference in *cspA* expression between wild-type and any of the mutant strains (Fig. 4D), the expression of that gene was lower (*P* < 0.01) in the absence of HOSCN in the ∆*rclB* mutant than in either the ∆*rclA* or ∆*rclC* mutants. The mechanisms and functions of the proteins encoded by the *csp* genes are not generally well understood (51), and the physiological relevance of this observation, if any, is unclear.

Perhaps, the most interesting to us was the very small set of genes whose expression did differ among the various *rcl* mutant strains. Two that stood out were *bhsA* and *ompC* (Fig. 4E and F). The *bhsA* gene encodes a small periplasmic protein of the same DUF1471 family as RclB, has been shown to affect both biofilm formation and cell envelope permeability to copper (57, 58), and was the only gene regulated by treatment with HOSCN that was more significantly upregulated (*P* < 0.0001) in the triple knockout of *rclABC* than in the single mutants (*P* < 0.001)(Fig. 4E; Dataset S1). This already suggested to us that there might be a link between cell envelope permeability, HOSCN treatment, and the function(s) of the *rcl* operon. Complementation experiments showed that ectopic expression of *bhsA* had a noticeable negative effect on the growth of ∆*rclB E. coli* even in the absence of stress, but did also generally increase its sensitivity to HOSCN (Fig. S5). The toxicity of *bhsA* expression, however, makes the specificity of this result difficult to determine, but it does not appear that BhsA can replace RclB for HOSCN defense.

Meanwhile, *ompC* encodes one of the major outer membrane porins (OMPs) of *E. coli* (75) and had a very striking and unique pattern of expression. Transcription of *ompC* was significantly (*P* < 0.05) induced by HOSCN in wild-type, ∆*rclA*, and ∆*rclABC* strains, but not in either of the ∆*rclB* or ∆*rclC* mutants (Fig. 4F; Dataset S1). The genetic regulation of the OMPs is complicated and affected by over a dozen known transcriptional regulators (76), but in general, *ompC* transcription increases in the presence of stressors affecting the cell envelope. This, therefore, suggested a model in which HOSCN might accumulate in the periplasmic space of wild-type or ∆*rclA* mutant strains (damaging the cell envelope and inducing *ompC* expression) but is freely permeable through the cell envelope of mutants lacking RclB or RclC. In the presence of cytoplasmic RclA (20), HOSCN would be rapidly degraded (preventing envelope damage and *ompC* induction). However, according to this model, in the mutant lacking all three Rcl proteins, HOSCN might be relatively stable and be able to react with and damage the cell envelope, restoring *ompC* induction in the ∆*rclABC* strain (Fig. 4F). We therefore turned our attention to the potential role of outer membrane and cell envelope permeability in *E. coli* resistance to HOSCN.

### Mutations in outer membrane porins protect *E. coli* from HOSCN

**Fig 5:**
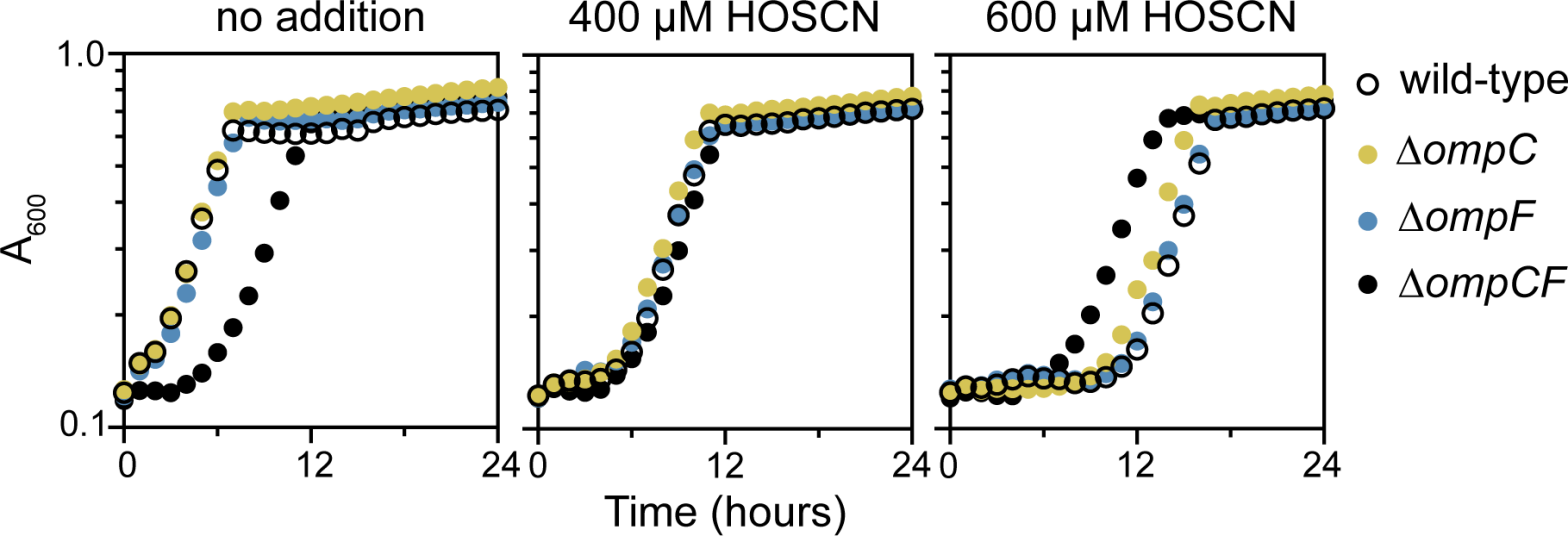
Mutations in *ompC* and *ompF* protect *E. coli* against HOSCN stress. *E. coli* MG1655 wild-type, MJG2411 (MG1655 ∆*ompC*), MJG2412 (MG1655 ∆*ompF*), and MJG2429 (MG1655 ∆*ompC* ∆*ompF::kan*^+^) were inoculated into M9 minimal medium containing the indicated concentrations of HOSCN and incubated at 37°C with shaking for 24 hours in a Tecan Sunrise plate reader, measuring A_600_ every 30 minutes. Each graph shows the mean of four technical replicates, and two additional experimental replicates are shown in Fig. S2.

OmpC, OmpF, and OmpA are the major OMPs in *E. coli* (75, 76). The loss of outer membrane permeability via OMP regulation has often been associated with antibiotic resistance in diverse bacteria (77), and deletions of either *ompC* or *ompF* are reported to protect *E. coli* against the HOSCN-generating enzyme LPO (78). We therefore made deletions of both *ompC* and *ompF* in our *E. coli* strain background to test those mutants’ sensitivity to HOSCN treatment (Fig. 5; Fig. S6). Single mutants lacking either *ompC* or *ompF* did not have any substantial sensitivity or resistance to HOSCN and grew equally well to the wild-type at moderate HOSCN concentrations (400 or 600 µM). Both ∆*ompC* and ∆*ompF* mutants were slightly more resistant to 800 µM HOSCN than the wild-type strain (Fig. S6). As expected, due to the loss of most of the porins allowing solute transfer across the outer membrane (75), the Δ*ompCF* double knockout grew more slowly without stress compared to the other strains, but it also displayed strong resistance to HOSCN. At higher HOSCN concentrations (600 or 800 µM), the double-knockout strain grew better than the wild-type or the single knockout strain (Fig. 5; Fig. S6). These results confirmed that reduction of outer membrane permeability by disrupting OMP function can confer HOSCN resistance in *E. coli*.

### RclB is homologous to other small periplasmic stress response proteins

**Fig 6:**
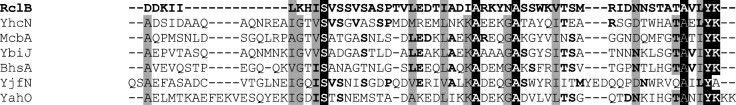
Alignment of RclB with other *E. coli* DUF1471 proteins. The EMBL MUSCLE multiple alignment tool (83) was used to compare RclB to the six most similar of the other DUF1471 proteins in *E. coli* MG1655 (59) (see Table S2 for additional information about these and the other five DUF1471 domains encoded by *E. coli* MG1655).

RclB belongs to a family of small proteins called the DUF1471 family, which are small periplasmic proteins exclusive to the *Enterobacteriaceae*, and only a few of which have any functional annotation (64). *E. coli* MG1655 encodes 10 DUF1471 paralogs (Table S2) (59). The amino acid sequences of the most similar paralogs to RclB are shown in Fig. 6. Most of these have no known functions, but notable among them are BhsA*,* discussed above (Fig. 4E; Fig. S5), and YjfN, which uses a hydrophobic alanine on its C-terminus to interact with the protease DegP, directing the degradation of OmpA under certain envelope stress conditions (79). BhsA is known to be associated with the outer membrane, where it reduces the permeability of the cell envelope to copper and therefore increases the resistance of *E. coli* to copper stress (57), but the exact mechanism by which it affects the permeability is not known. Some of the other DUF1471 proteins are also associated with specific stress responses (e.g., YhcN with oxidative stress [80], McbA with biofilm formation [81], or YahO with radiation resistance [82]), but nothing is known about how they contribute to these responses.

**Fig 7:**
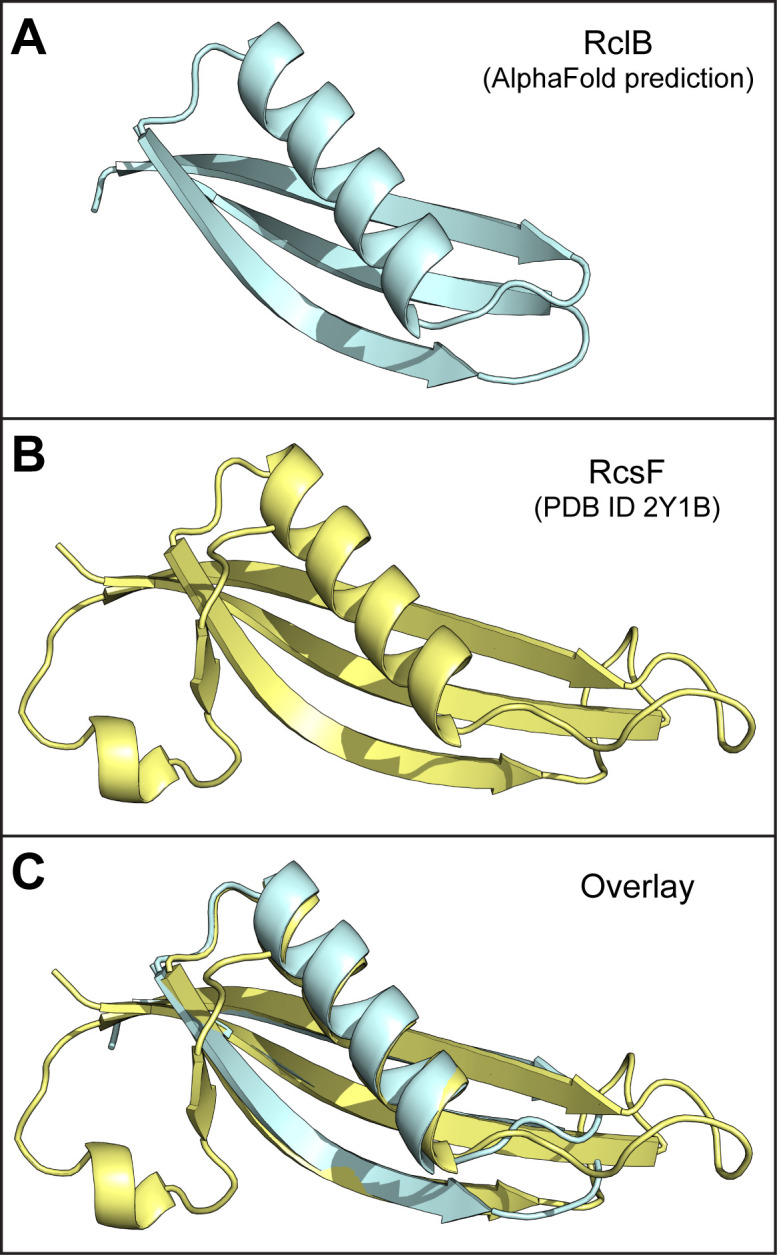
RclB is a structural homolog of the OMP-interacting lipoprotein RcsF. (**A**) The AlphaFold prediction of the structure of RclB (86, 87) and (**B**) the crystal structure of RcsF (88) were identified as structural homologs (**C**) using COFACTOR (84), which calculated an RMSD^a^ score = 1.47 Å. Protein structures were visualized using Pymol.

A separate search for structural homologs of RclB, using the COFACTOR tool (84), identified the *E. coli* RcsF protein as containing a highly similar three-dimensional structure (RMSD^a^ = 1.47 Å) (Fig. 7). RcsF is a sensor lipoprotein that forms a complex with OmpC, OmpF, or OmpA (called the RcsF-OMP complex), inserting into the β-barrel pores of those OMPs and using its surface-exposed lipidated loop to detect membrane damage (85). RcsF is required for activation of the envelope stress-responsive Rcs system (85). While RclB is not very similar to RcsF at the primary sequence level (18% identical, 30% similar; Table S2) and does not possess the loop that forms the lipidated section of RcsF (Fig. 7C, lower left corner), the structural homology could still indicate some sort of interaction with OMPs, and indeed, based on this homology, we hypothesized that interaction with or regulation of OMPs could potentially be a common mechanism for all of the stress-responsive DUF1471 proteins (Fig. 6; Table S2).

### Neither HOSCN treatment nor mutation of *rclB* and *rclC* has any effect on the abundance of OMPs

**Fig 8:**
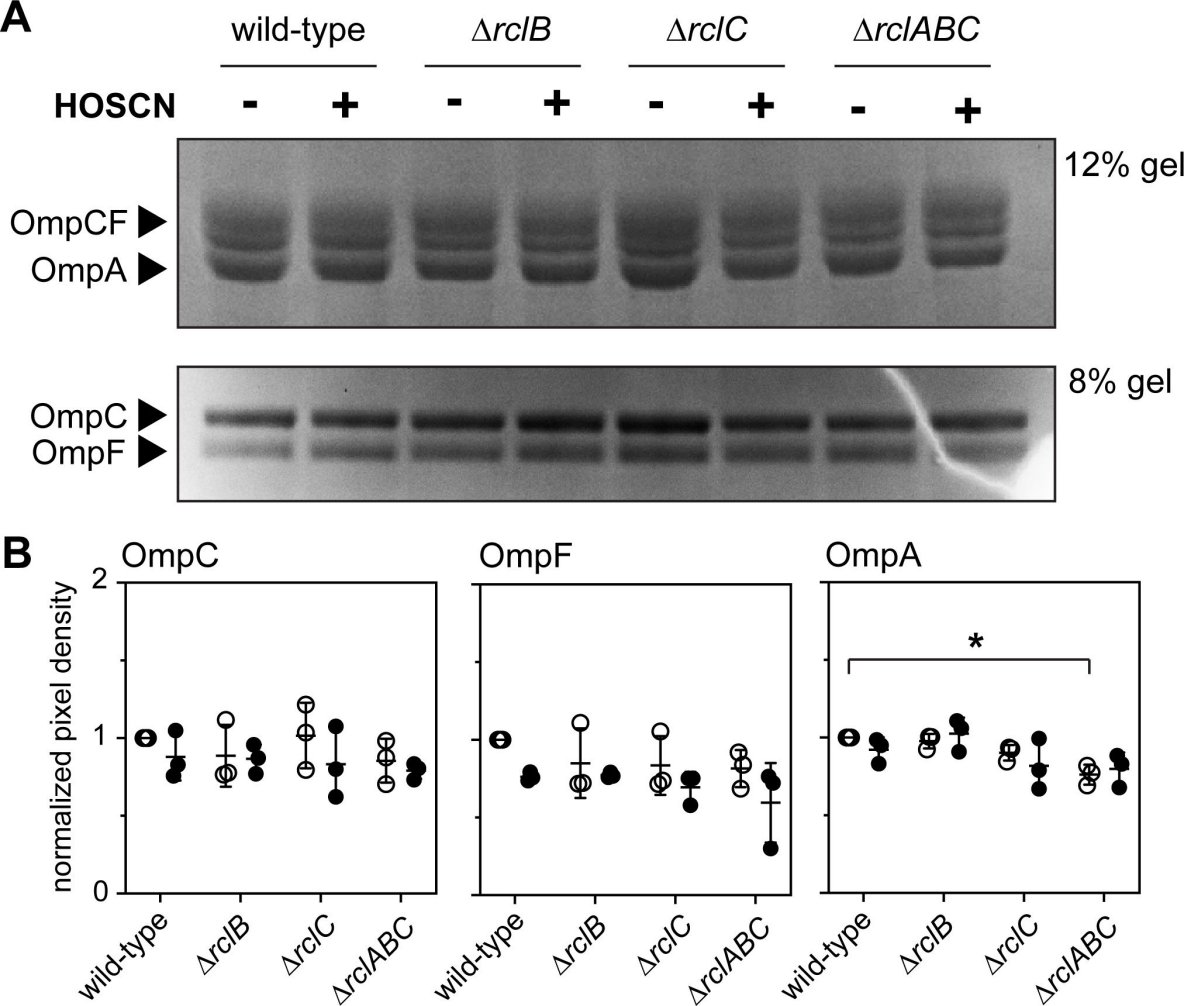
HOSCN stress does not have major impacts on the abundance of OmpC, OmpF, or OmpA in *E. coli*. *E. coli* MG1655 wild-type, MJG0047 (MG1655 ∆*rclB*), MJG0013 (MG1655 ∆*rclC::cat*^+^), and MJG0901 (MG1655 ∆*rclABC*) were grown at 37°C with shaking to A_600_ = 0.3 in M9 minimal medium, and then 200 µM HOSCN was added to treatment samples. Cultures were incubated at 37°C with shaking to A_600_ = 1.2–1.3, and then outer membrane protein fractions were isolated and separated on (**A**) 12% or 8% acrylamide Bolt SDS-PAGE gels (Invitrogen) and visualized by Coomassie Blue staining. Complete gel images from triplicates of this experiment are shown in Fig. S3. (**B**) The indicated protein band intensities were measured with ImageJ (89) and normalized to their intensities in untreated wild-type *E. coli* for each experiment. Statistical significance calculated in Prism GraphPad by two-way ANOVA: * =*P*-value <0.05. Full ANOVA results in Table S1.

Since ∆*ompCF* mutants are resistant to HOSCN (Fig. 5) and RclB is homologous to two proteins known to interact with or regulate OMPs at the post-transcriptional level (i.e., YjfN and RcsF), we tested whether either exposure to HOSCN or *rcl* mutations affected the abundance or ratio of the OmpC, OmpF, or OmpA proteins in *E. coli* (75, 76). Under our growth conditions, OmpC, OmpF, and OmpA were all highly abundant (Fig. 8A; Fig. S7). We were unable to consistently isolate high-quality outer-membrane fractions from *E. coli* exposed to high HOSCN concentrations (possibly supporting the idea that HOSCN has important impacts on the outer membrane), but exposure to 200 µM HOSCN (sufficient to strongly induce transcription of the *rclABC* operon) (20) had no significant effect on the abundance of any of the OMPs (Fig. 8B). The ∆*rclABC* mutant had slightly less OmpA than the wild-type (~80%, *P* < 0.05) under both non-stress and HOSCN treatment conditions (Fig. 8B), but the effect was small, and the physiological relevance of this result is unclear. Based on these results, we conclude that neither RclB nor RclC is likely to function by regulating the bulk synthesis or degradation of any of the major OMPs, but this does not preclude the possibility that they interact with one or more of those proteins or with other, less abundant OMPs to regulate their functions or the ability of HOSCN to diffuse through their pores.

### Deletion of *rcl* genes does not sensitize the *E. coli* periplasm to oxidation by HOSCN

**Fig 9:**
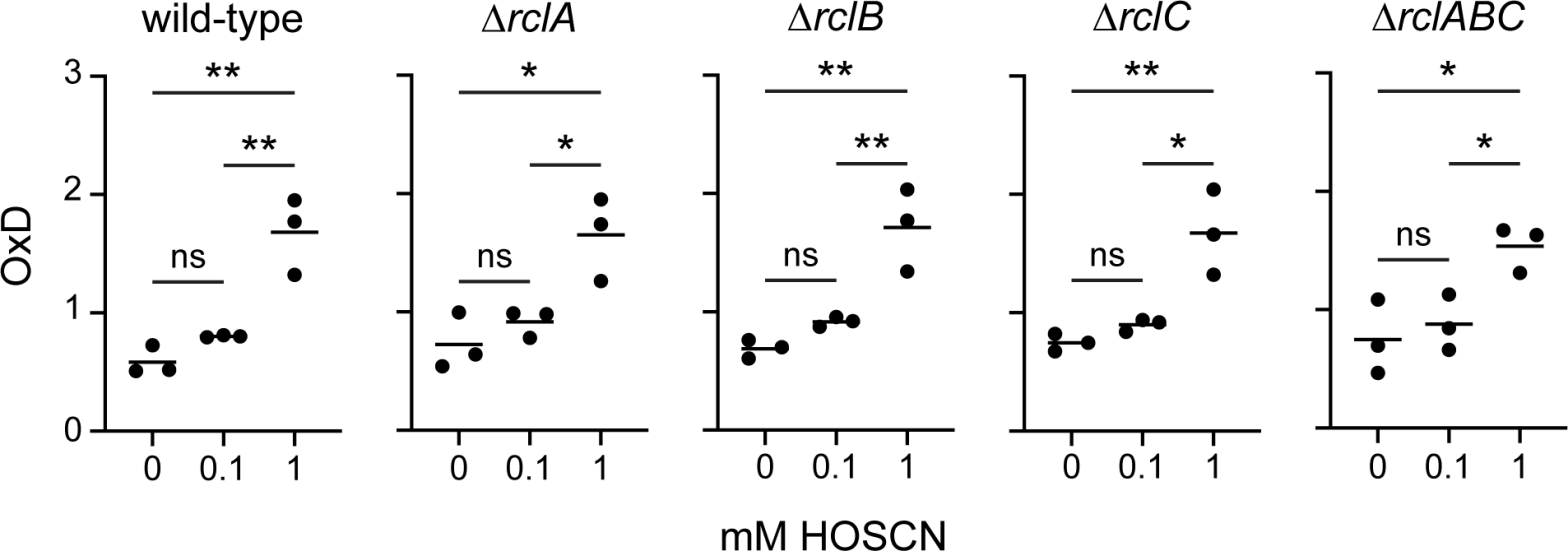
Deletion of *rcl* genes does not make the periplasm of *E. coli* more sensitive to oxidation by HOSCN. *E. coli* MG1655 wild-type, MJG0047 (MG1655 ∆*rclB*), MJG0013 (MG1655 ∆*rclC::cat*^+^), and MJG0901 (MG1655 ∆*rclABC*) containing plasmid pPT_roGFP2-iL were exposed to the indicated concentrations of HOSCN, and the relative oxidation of the periplasm (OxD) was calculated as previously described (90). Statistical significance calculated in Prism GraphPad by one-way ANOVA: ns = not significant, * =*P*-value <0.05; ** =*P*-value <0.01. Full ANOVA results are given in Table S1.

To more directly test whether RclB functions by reducing the ability of HOSCN to penetrate the outer membrane, we used a ratiometric fluorescence reporter of periplasmic oxidation (90) to measure the impact of HOSCN on the redox state of the periplasm. This experiment showed that treatment with 1 mM HOSCN did significantly (*P* < 0.05–0.01) oxidize the periplasm of *E. coli* but found no differences in this impact between wild-type cells and ∆*rclA*, ∆*rclB*, ∆*rclC*, or ∆*rclABC* mutants (Fig. 9), arguing against the hypothesis that any element of the RclABC defense system acts specifically to shield the periplasm against oxidation by HOSCN.

### Conclusions

#### RclABC as a coordinated HOSCN defense system in *E. coli*

In this study, we have characterized the overall transcriptomic response of *E. coli* MG1655 to treatment by HOSCN for the first time. We observed that *E. coli* has a more specific response to HOSCN than what has been shown in other immune-derived oxidants, with many of the typical oxidative stress responses being only partially upregulated (Fig. 1). When any of the *rcl* genes were mutated, *E. coli* demonstrated both greater sensitivity (Fig. 2) and a greater transcriptional response to HOSCN (Fig. 3 and 4), although surprisingly, the transcriptional response to HOSCN was mostly highly comparable in all knockout strains and not dependent on one *rcl* gene over the other. Transcriptional signatures (Fig. 4, 6 and 7) lead us to suggest a model in which RclA, RclB, and RclC act as a coordinated HOSCN defense system, with RclB and RclC likely acting together, based on the similarity of the phenotypes of ∆*rclB* and ∆*rclC* mutants (Fig. 2 and 4F). We hypothesized based on the structural and sequence homology to other proteins that RclB might do this by interaction with OMPs (Fig. 6 and 7), but deletion of *rclB* did not notably change the abundance of OmpA, OmpC, or OmpF (Fig. 8), nor did it sensitize the periplasm to HOSCN oxidation (Fig. 9), arguing against this model. The molecular mechanism(s) by which RclB and the inner membrane protein RclC protect against HOSCN therefore remain to be determined.

## MATERIALS AND METHODS

### Bacterial strain construction

All bacterial strains used in this study can be found in Table 1. All strains and plasmids generated in the course of this work are available from the corresponding author upon request. The *E. coli* Δ*ompC* and Δ*ompF* strains were generated by transducing the Δ*ompC768::kan^+^* and Δ*ompF746::kan^+^* alleles from the Keio collection (91) into MG1655 (92) using P1*vir* phage (93). This generated strains MJG2366 and MJG2367, respectively. The kanamycin resistance cassette in both strains was resolved (94) to yield strains Δ*ompC768* (MJG2411) and Δ*ompF746* (MJG2412). The double-knockout strain MJG2429 (Δ*ompC768*Δ*ompF746::kan^+^*) was generated by transducing the Δ*ompF746::kan^+^* + into MJG2411 using P1*vir*. Plasmids were transformed into *E. coli* strains by electroporation (95). When appropriate, kanamycin (25 or 50 µg mL^−1^) or ampicillin (100 µg mL^−1^) was added to bacterial growth media.

**TABLE 1:** Strains and plasmids used in this study^*a*^

Strain	Marker	Genotype	Reference
MG1655		F^-^, λ^-^, *rph-1 ilvG^-^ rfb-50*	(92)
MJG0013	Cm^R^	MG1655 Δ*rclC745::cat^+^*	(38)
MJG0047		MG1655 Δ*rclB746*	(38)
MJG1958		MG1655 *∆rclA747*	(20)
MJG0901		MG1655 *∆rclABC*	(37)
MJG2366	Kn^R^	MG1655 Δ*ompC768*::*kan^+^*	
MJG2367	Kn^R^	MG1655 Δ*ompF746*::*kan^+^*	
MJG2411		MG1655 Δ*ompC768*	
MJG2412		MG1655 Δ*ompF746*	
MJG2429	Kn^R^	MG1655 Δ*ompC768 ∆ompF746*::*kan^+^*	
Plasmid			
pPT_roGFP2-iL	Ap^R^	P*_tac_*::*torA*(signal sequence)*-roGFP2-iL^+^ bla* +	(90)
pUC18	Ap^R^	*bla* ^+^	(96)
pBHSA1	Ap^R^	*bhsA* ^+^ *bla* ^+^	
pRCLB3	Ap^R^	*rclB* ^+^ *bla* ^+^	

^*a*^ Unless otherwise indicated, strains and plasmids were generated in the course of this work. Abbreviations: Ap^R^, ampicillin resistance; Cm^R^, chloramphenicol resistance; Kn^R^, kanamycin resistance.

### Plasmid construction

The coding domain sequences of *bhsA* and *rclB* from *E. coli* MG1655 (locus tags b1112 and b0303, respectively) were synthesized by GenScript between the *Kpn*I and *Bam*HI sites of plasmid pUC18 (96), yielding plasmids pBHSA1 and pRCLB3.

### RNA sequencing

For RNA extraction, single colonies of *E. coli* were inoculated into 5 mL of LB and grown overnight at 37°C with shaking. The next day, *E. coli* was subcultured into M9 minimal media containing 100 µM FeCl_3_ and grown to the mid log phase (absorbance at 600 nm = 0.4–0.5). Cultures were treated with 600 µM HOSCN for 15 minutes, at which point they were harvested, and RNA was extracted using the Qiagen RNeasy PowerMicrobiome Kit. mRNA sequencing was carried out by SeqCenter (Pittsburgh, PA). Samples were DNAse-treated with Invitrogen DNAse (RNAse free). Library preparation was performed using Illumina’s Stranded Total RNA Prep Ligation with Ribo-Zero Plus kit and 10 bp unique dual indices (UDI). Sequencing was done on a NovaSeq X Plus, producing paired-end 150 bp reads. Demultiplexing, quality control, and adapter trimming were performed with bcl-convert (v4.1.5). Quality control and adapter trimming were performed with bcl-convert. Read mapping was performed with HISAT2 (97). Read quantification was performed using Subread’s featureCounts functionality (98). Read counts loaded into R were normalized using edgeR’s Trimmed Mean of M values (TMM) algorithm. Subsequent values were then converted to counts per million (CPM). Differential expression analysis was performed using edgeR’s glmQLFTest.

### Quantitative reverse transcriptase PCR

RNA was purified from log-phase *E. coli* cells treated with 600 µM NaSCN (Thermo Fisher) for 15 minutes, using the Qiagen RNeasy Miniprep Kit (Qiagen) with a pre-treatment using the RNAprotect Bacteria Reagent (Qiagen). Complementary DNA (cDNA) was produced using the Omniscript Reverse Transcriptase Kit (Qiagen), and qRT-PCR was done in a Bio-Rad CFX96 Thermocycler. Expression of *cynT* was normalized against *rrsD* expression. Changes were calculated using the 2^-ΔΔCt^ method (99). The iQ SYBR Green Supermix (Bio-Rad) was used with *cynT* primers 5′ AAG CGG GAA GCC TTG TTT A 3′ and 5′ CAT ACT CCA CCG AAG CAG AAA 3′ and *rrsD* primers 5′ GAG CAA GCG GAC CTC ATA AA 3′ and 5′ TCC CGA AGG TTA AGC TAC CTA 3′.

### Measuring bacterial growth under HOSCN stress

Single colonies of *E. coli* were inoculated in M9 minimal media (100) containing 0.2% glucose and 100 µM FeCl_3_ and grown overnight at 37°C with shaking. The next day, cultures were harvested and normalized to OD_600_ = 0.05 in a 96-well plate with M9 minimal media containing the indicated concentrations of HOSCN. The plate was covered with a BreatheEasy plate film (Andwin Scientific) and placed in a Tecan Sunrise plate reader where the absorbance was measured at 600 nm every 15 minutes for 24 hours, with shaking in between each measurement. HOSCN was made and quantified fresh the day of the experiment, as previously described (20). Due to the highly reactive nature of HOSCN, the exact period of growth inhibition caused by a given dose of HOSCN varied from day to day, so representative growth curve data are shown in Fig. 2 and 5, with data from additional independent experimental replicates shown in Fig. S3 and S5, respectively, to illustrate the consistency in the relative sensitivity of mutant strains to the wild-type and to one another.

### Structural homology

Structural homology of RclB was investigated with the COFACTOR tool, using the default parameters (84). The pdb file of the predicted structure of RclB was obtained from AlphaFold (86, 87). The ribbon structure images were produced using Pymol (The PyMOL Molecular Graphics System, Version 3.0 Schrödinger, LLC.).

### Cell collection for isolation of outer membrane proteins

Single colonies of *E. coli* were inoculated into 5 mL of LB and grown overnight at 37°C with shaking. The next day, 500 µL of the overnight culture was subcultured into 25 mL of M9 minimal growth media and grown to early log phase (absorbance at 600 nm = 0.3), at which point treatment samples were dosed with HOSCN to a final concentration of 200 µM and then continued to grow until the optical density reached 1.2–1.3. Cell pellets were spun-down and stored at −80°C until needed.

### Outer membrane protein preparation

Outer membrane fractions were prepared by a modification of a previously published method (101). Frozen cell pellets were thawed at room temperature and then resuspended in PBS. Cultures were sonicated to break open cells for 2 minutes, 5 seconds on and 5 seconds off, at 50% amplification, 50 V, using a Model 120 Sonic Dismembrator (Fisher). Samples were then centrifuged at 1,400 x *g* for 10 minutes to collect the cell debris. Supernatants were centrifuged at 85,000 x *g* for 40 minutes in a Beckman-Coulter Optima XPN-80 ultracentrifuge and then solubilized in a solution of 2% Triton-X 100 in Tris-HCl (pH 7.5) for 30 minutes at 37°C. Outer membranes were then collected by ultracentrifugation at 85,000 x *g* for 40 minutes. Proteins were visualized by running on 8% or 12% Invitrogen Bolt gels with MOPS buffer and staining with Coomassie Blue, and the band intensity was quantified with ImageJ (89).

### Measurement of periplasmic oxidation

The relative oxidation of the periplasm was measured using plasmid pPT_roGFP2-iL, as previously described (90). Overnight cultures of *E. coli* were subcultured into M9 minimal media with 0.2 mM IPTG and grown to mid log-phase (OD_600_ = 0.3). Cultures were harvested and washed once with phosphate buffer solution (PBS) and normalized to OD_600_ = 1 in PBS containing 1 mM 2,2-dipyridyl disulfide 98% (Fisher Scientific AAA1111806) as the oxidation control, 10 mM Dithiothreitol (DTT) (Gold Biotechnics DTT100), or HOSCN. Hundred microliters of the treated cultures was transferred to a black, clear-bottom 96-well plate (Zellkultur microplate greiner bio-one 655090), and fluorescence intensity was measured at excitation wavelengths 395 and 465 nm and emission wavelength of 525 nm in a Spark plate reader (Tecan). The probe’s oxidation (OxD) was calculated as (R – R_red_) / ((I_465_ox / I_465_red) x (R_ox_ – R) + (R – R_red_)), where R = the ratio of fluorescence emission intensities at 395 and 465 nm, R_red_ = the 395/465 ratio of fully reduced samples, R_ox_ = the 395/465 of fully oxidized samples, and I_465_ox and I_465_red are the fluorescence intensities at 465 nm under fully oxidized or fully reduced conditions, respectively (90).

## Data Availability

The raw data for the transcriptomic experiment described above are available in the NIH Sequence Read Archive (accession number PRJNA989513), and a human-readable version (with CPMs converted to CPM + 1 to allow for calculation of the log_2_-fold change in expression for all genes, including those with no detectable reads under some conditions) is available as Dataset S1. All other raw data are available on FigShare (DOI: 10.6084 /m9.figshare.c.7742993).

## SUPPLEMENTAL MATERIAL

Dataset S1 jb.00524-24-s0001.xlsx RNA sequencing results of HOSCN-treated *E. coli*.

Supplemental figures and tables jb.00524-24-s0002.pdf Figures S1 to S7, and Table S2.

Table S1 jb.00524-24-s0003.xlsx Two-way ANOVA analysis of gene expression and 43 protein abundance data in Figures 1, 4, 8, and 9 complete results.

ASM does not own the copyrights to Supplemental Material that may be linked to, or accessed through, an article. The authors have granted ASM a non-exclusive, world-wide license to publish the Supplemental Material files. Please contact the corresponding author directly for reuse.
