# Supplementary material for: Genome-wide characterization of hypothiocyanite stress response in Escherichia coli
Source: J Bacteriol. 2025 Apr 29;207(5):e00524-24. doi: 10.1128/jb.00524-24 (PMC12096828; doi:10.1128/jb.00524-24)
Supplement: Supplemental figures and tables — Figures S1 to S7, and Table S2. [file jb.00524-24-s0002.pdf]

SUPPLEMENTAL INFORMATION

Genome-wide characterization of hypothiocyanite stress response in *Escherichia coli*

Julia D. Meredith [a], Michael J. Gray [a, #]

[a] Department of Microbiology, School of Medicine, University of Alabama at Birmingham, Birmingham, AL 35294

Running Head: The genetic response of *E. coli* to hypothiocyanite

[#] Address correspondence to Michael J. Gray, mjgray@uab.edu

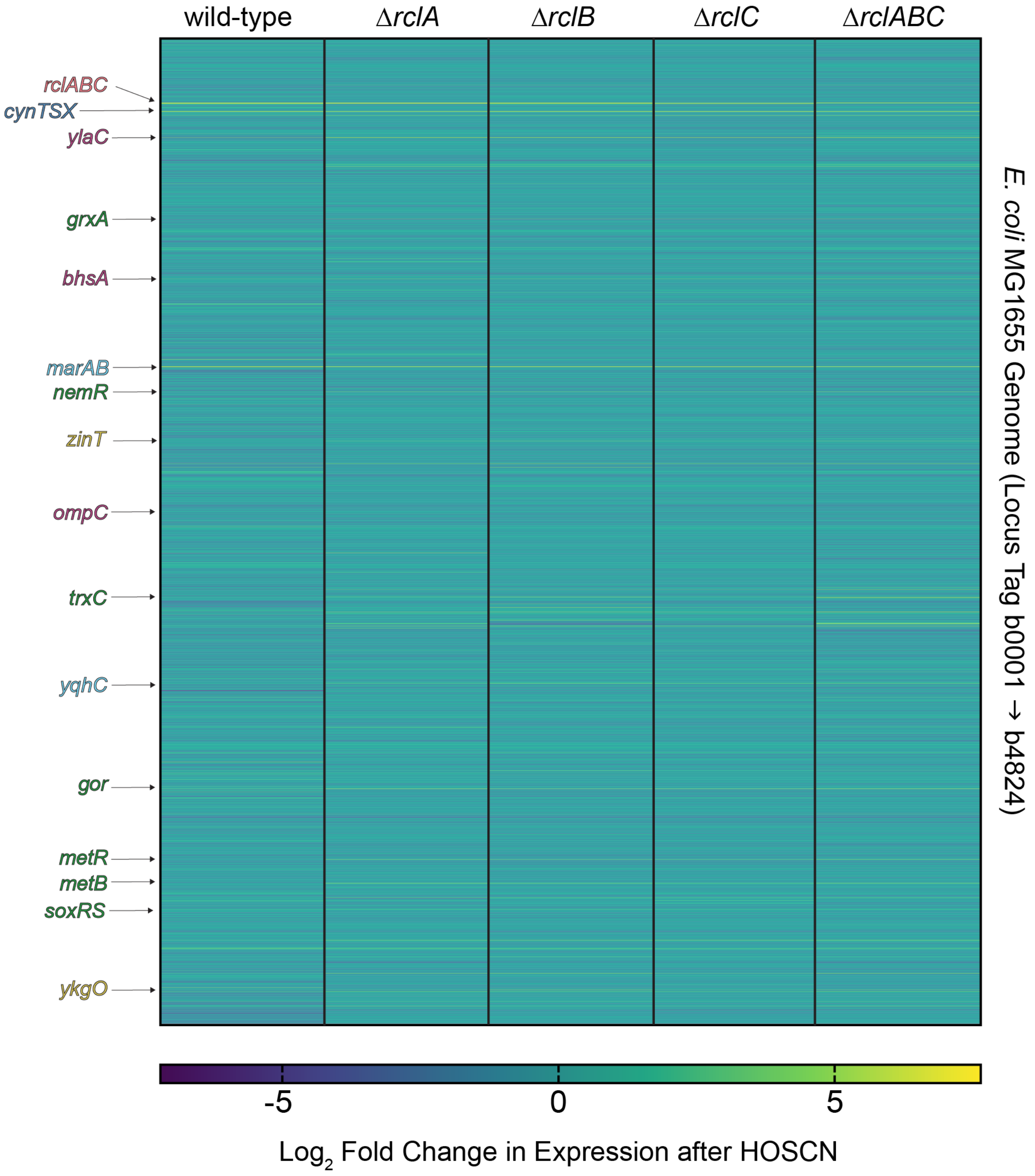

SUPPLEMENTAL FIGURE S1 Heatmap of the *E. coli* transcriptomic response to HOSCN. (A different visualization of the data shown in **FIGS 1** and **3**.) The *E. coli* transcriptional response to 600 μM HOSCN in strains MG1655 (wild-type), MJG1958 (MG1655 Δ*rclA*), MJG0047 (MG1655 Δ*rclB*), MJG0013 (MG1655 Δ*rclC*::*cat*$^{+}$) or

MJG0901 (MG1655 Δ*rclABC*), represented as a heatmap. Full RNA-seq results are in **SUPPLEMENTAL DATA SET 1**.

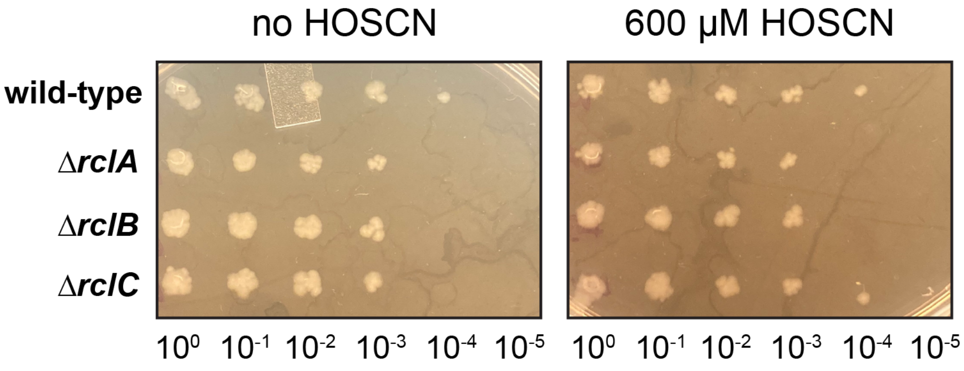

SUPPLEMENTAL FIG S2 600 μM HOSCN does not kill *E. coli*. *E. coli* MG1655 wild-type, MJG1958 (MG1655 Δ*rclA*), MJG0047 (MG1655 Δ*rclB*), and MJG0013 (MG1655 Δ*rclC*::*cat*$^{+}$), were inoculated into M9 minimal medium containing 600 μM HOSCN, incubated 15 min at 37°C, and then 10-fold serial dilutions were spotted on LB agar and incubated overnight at 37°C before imaging.

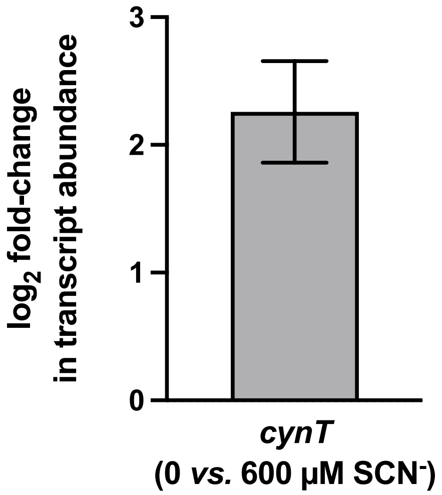

SUPPLEMENTAL FIG S3 SCN^- induces expression of *cynT*. qRT-PCR was performed using *cynT*-specific primers on RNA isolated from wild-type *E. coli* after exposure to 600 µM SCN^- for 15 minutes (n=2, error bars=range).

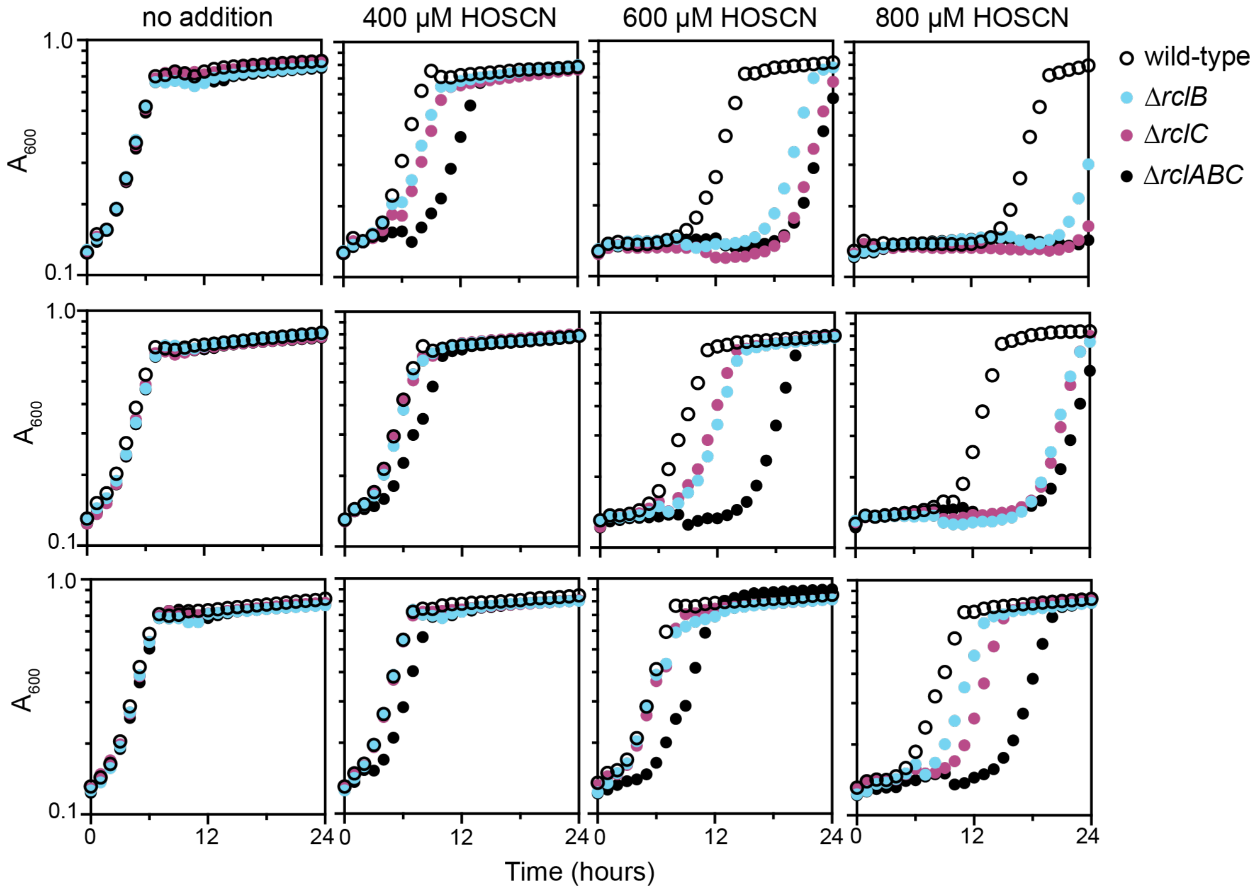

SUPPLEMENTAL FIG S4 RclB and RclC are required for HOSCN stress resistance in *E. coli*. (Triplicates of the experiment shown in **FIG 2**.) *E. coli* MG1655 wild-type, MJG0047 (MG1655 Δ*rclB*), MJG0013 (MG1655 Δ*rclC*::*cat*$^+$), and MJG0901 (MG1655 Δ*rclABC*) were inoculated into M9 minimal medium containing the indicated concentrations of HOSCN and incubated at 37°C with shaking for 24 hours in a Tecan Sunrise plate reader, measuring A_{600} every 30 minutes. Each graph shows the mean of four technical replicates.

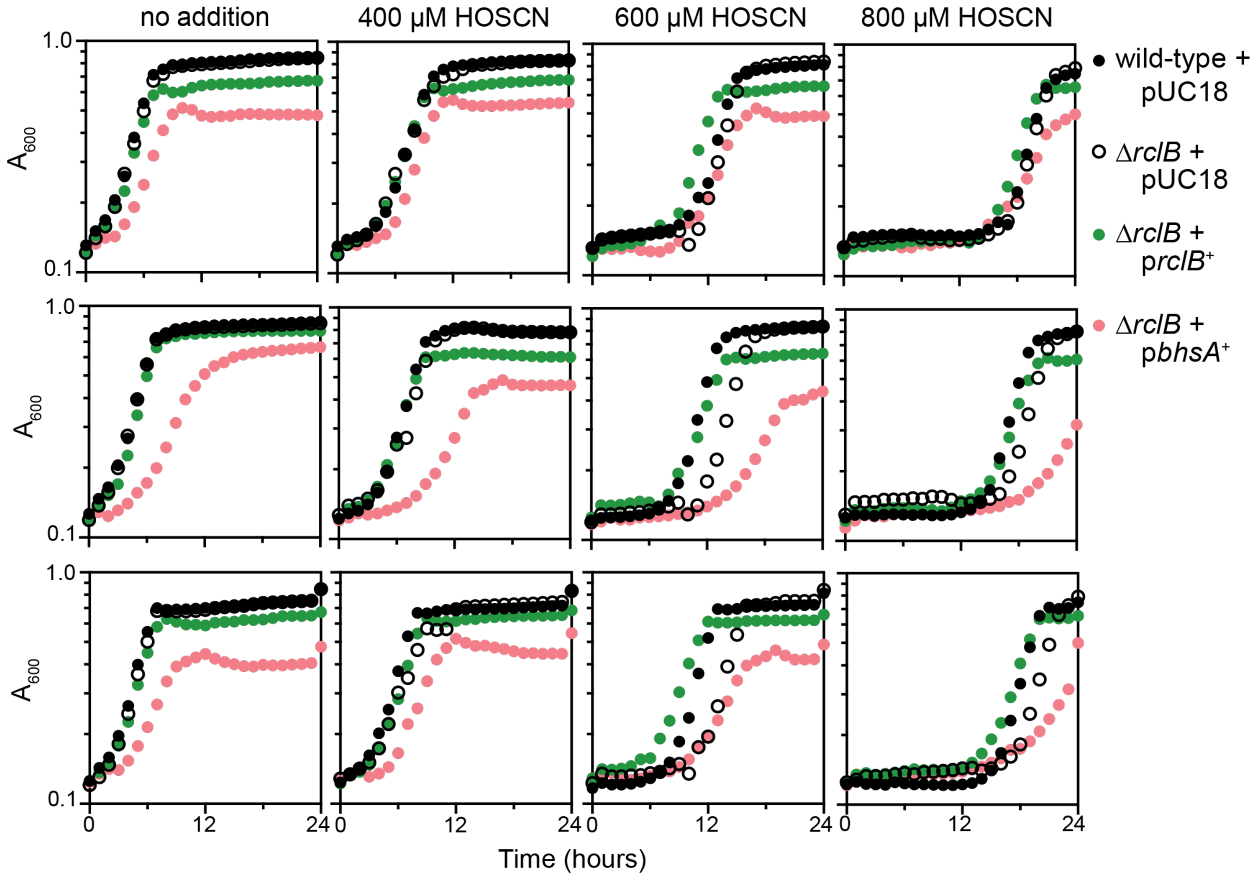

SUPPLEMENTAL FIG S5 Ectopic expression of *bhsA* does not protect *E. coli* against HOSCN. *E. coli* MG1655 wild-type or MJG0047 (MG1655 Δ*rclB*) containing the indicated pUC18-derived plasmids (p*rclB*$^+$ = pRCLB3; p*bhsA*$^+$ = pBHSA1) were inoculated into M9 minimal medium containing the indicated concentrations of HOSCN and incubated at 37°C with shaking for 24 hours in a Tecan Sunrise plate reader, measuring A_{600} every 30 minutes. Each graph shows the mean of two technical replicates.

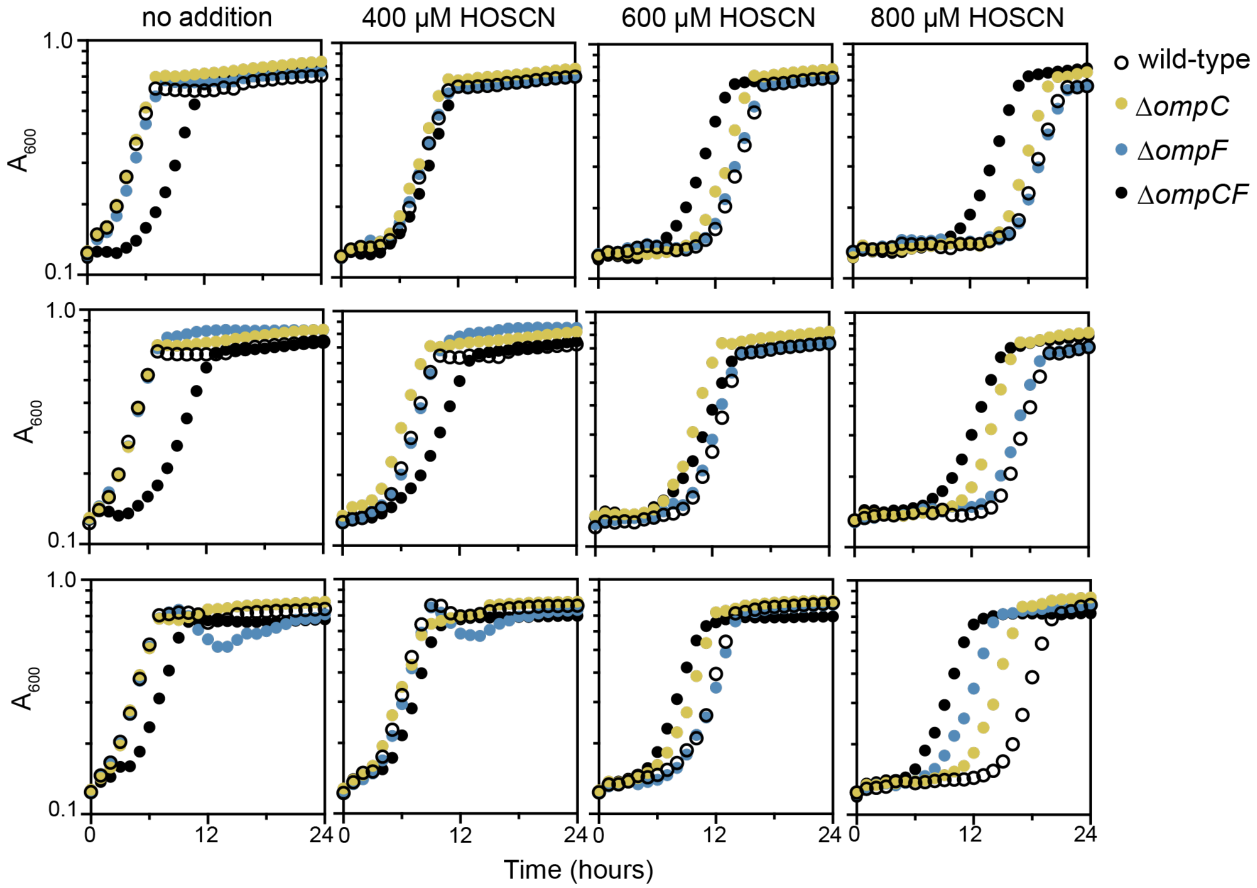

SUPPLEMENTAL FIG S6 Mutations in *ompC* and *ompF* protect *E. coli* against HOSCN stress. (Triplicates of the experiment shown in **FIG 5**.) *E. coli* MG1655 wild-type, MJG2411 (MG1655 Δ*ompC*), MJG2412 (MG1655 Δ*ompF*), and MJG2429 (MG1655 Δ*ompC* Δ*ompF*::*kan*$^+$) were inoculated into M9 minimal medium containing the indicated concentrations of HOSCN and incubated at 37°C with shaking for 24 hours in a Tecan Sunrise plate reader, measuring A_{600} every 30 minutes. Each graph shows the mean of four technical replicates.

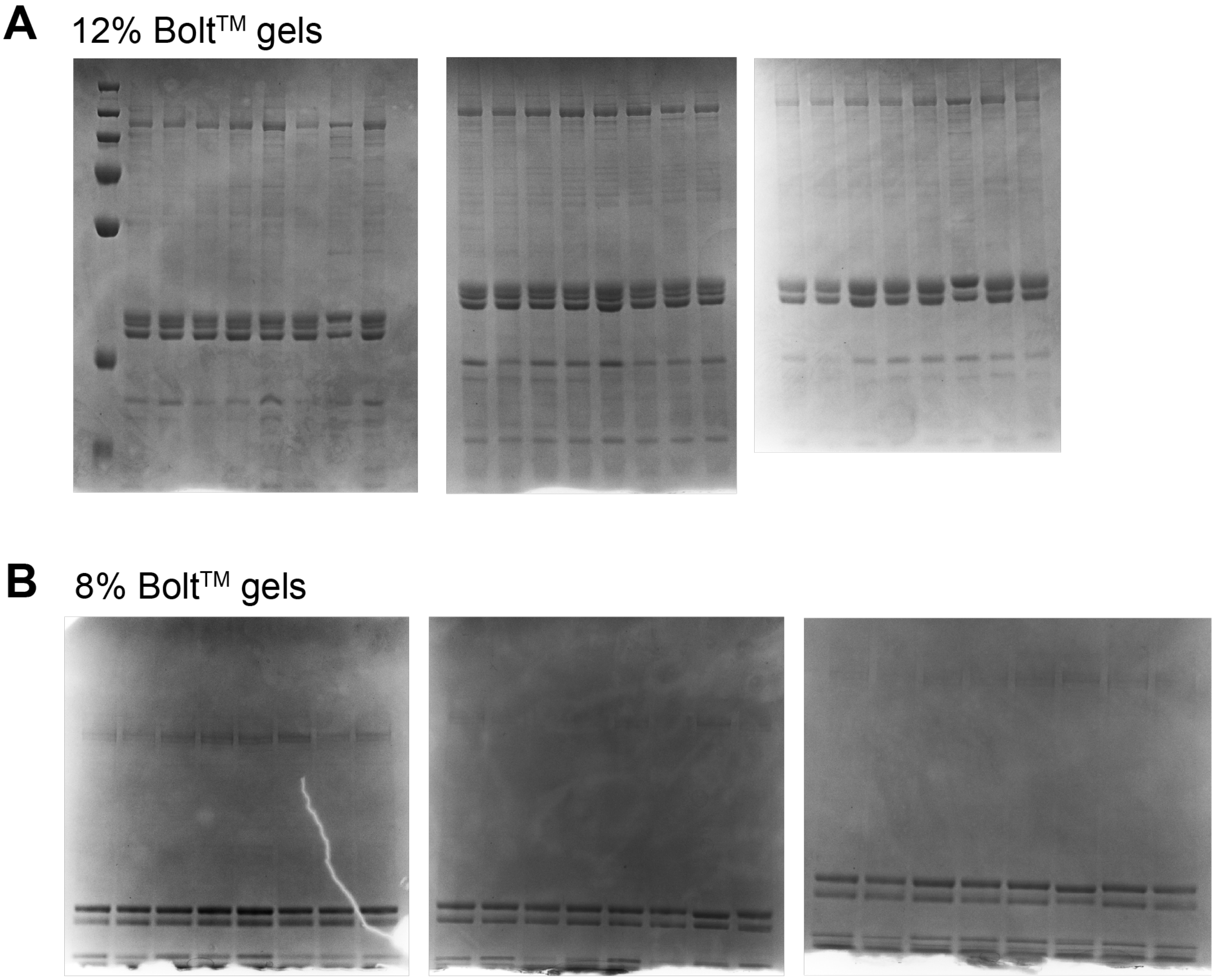

SUPPLEMENTAL FIG S7 HOSCN stress does not have major impacts on the abundance of OmpC, OmpF, or OmpA in *E. coli*. (Complete gel images from triplicates of the experiment shown in **FIG 8**.) *E. coli* MG1655 wild-type, MJG0047 (MG1655 Δ*rclB*), MJG0013 (MG1655 Δ*rclC*::*cat*$^{+}$), and MJG0901 (MG1655 Δ*rclABC*) were grown at 37°C with shaking to A_{600} = 0.3 in M9 minimal medium, then 200 μM HOSCN was added to treatment samples. Cultures were incubated at 37°C with shaking to A_{600} = 1.2 – 1.3, then outer membrane protein fractions were isolated and separated on (**A**) 12% or (**B**) 8% acrylamide Bolt™ SDS-PAGE gels (Invitrogen) and visualized by Coomassie Blue staining.

SUPPLEMENTAL TABLE S1 Two-way ANOVA analysis of gene expression, protein abundance, and periplasmic oxidation data in FIGS 1, 4, 8, and 9, complete results. Two-way ANOVA was used to compare all treatments (GraphPad Prism 10). All CPM+1 values used to generate **FIGS 1** and **4** (see **SUPPLEMENTAL DATA SET 1**) were $\log_{10}$-transformed before statistical analysis.

SUPPLEMENTAL TABLE S2 *E. coli* RclB homologs. The EMBL EMBOSS Needle pairwise alignment tool (1) was used to compare RclB to each of the other DUF1471 proteins in *E. coli* MG1655 (2)(see **FIG 6**), plus the structural homolog RcsF (see **FIG 7**). Note that YdgH contains three DUF1471 domains, each of which is indicated by its amino acid position within that protein and was compared to RclB separately.

Protein	% Identity	% Similarity	Score	function
RclB	100	100	378	hypothiocyanite resistance protein
YhcN	28	49	80	involved in hydrogen peroxide, cadmium, and acid stress response
McbA	31	52	76	modulates biofilm formation
YbiJ	28	43	69	unknown
BhsA	29	52	64	modulates outer membrane permeability in response to copper stress
YjfN	26	43	49	suicide activator of DegP protease, promotes OmpA degradation
YahO	21	41	45	involved in radiation resistance

YjfY	25	39	37	unknown
YdgH (259-314)	19	28	36	unknown
YdgH (119-174)	20	29	32	unknown
BsmA	25	40	28	lipoprotein involved in acid and peroxide stress resistance in biofilms
YdgH (35-90)	23	35	27	unknown
RcsF	18	30	17	lipoprotein that interacts with OmpA, OmpC, and OmpF to sense membrane damage

SUPPLEMENTAL REFERENCES

1. Madeira F, Madhusoodanan N, Lee J, Eusebi A, Niewielska A, Tivey ARN, Lopez R, Butcher S. 2024. The EMBL-EBI Job Dispatcher sequence analysis tools framework in 2024. Nucleic Acids Res 52:W521-W525.
2. Karp PD, Paley S, Caspi R, Kothari A, Krummenacker M, Midford PE, Moore LR, Subhraveti P, Gama-Castro S, Tierrafria VH, Lara P, Muniz-Rascado L, Bonavides-Martinez C, Santos-Zavaleta A, Mackie A, Sun G, Ahn-Horst TA, Choi

H, Covert MW, Collado-Vides J, Paulsen I. 2023. The EcoCyc Database (2023). EcoSal Plus 11:eesp00022023.
